# The Challenge of Managing Children With Periodic Fever Syndromes in the Era of COVID-19

**DOI:** 10.3389/fped.2020.620621

**Published:** 2021-01-06

**Authors:** Rita Consolini, Giorgio Costagliola, Marco Gattorno

**Affiliations:** ^1^Section of Clinical Immunology and Rheumatology, Division of Pediatrics, Department of Clinical and Experimental Medicine, University of Pisa, Pisa, Italy; ^2^Center for Autoinflammatory Diseases and Immunodeficiencies, Istituto di Ricovero e Cura a Carattere Scientifico (IRCCS) Giannina Gaslini, Genoa, Italy

**Keywords:** autoinflammatory diseases, biologic drug, coronavirus, corticosteroids, disease flare, nasal swab, school attendance, SARS-CoV-2

## Introduction

Coronavirus disease 2019 (COVID-19) pandemic has dramatically changed the diagnostic and therapeutic approach to children presenting with fever or signs of respiratory infection. This carries a significant impact on the quality of life of the children and their families and on the healthcare system, which has markedly increased following the school and childhood community reopening in autumn. Indeed, fever, cough and other signs of upper respiratory infection (rhinorrhea, pharyngitis) are the most common clinical manifestations of pediatric COVID-19, while the severe manifestations of the disease (pneumonia, systemic and multi-organ involvement) are evidenced only in a reduced percentage of cases (1). According to most national and international guidelines, the finding of fever is currently followed by measures to prevent the spreading of SARS-CoV-2 virus, including the isolation of the child and the execution of a rhino-pharyngeal swab for the detection of SARS-CoV-2 genome (2). Although recommendations are not univocal, in most of the countries the exclusion of COVID-19 (on the basis of the clinical assessment or, most frequently, after the execution of a swab) is also necessary for the reintroduction to school and community after an absence related to a febrile episode. As a consequence, during the pandemic, a single episode of fever can be responsible of a high rate of medicalization and stress for the child and the caregivers. Given the high frequency of respiratory morbidity of the childhood population during the autumn-winter season, also the number of days of school lost by a child, together with the days of work lost by the caregiver, can be consistent. The particular case of children with periodic fever syndromes (PFS) deserves a great attention, since they represent a population experiencing a high annual number of febrile episodes. In this work we discuss the clinical implications deriving from the pandemic for the diagnosis and management of children with PFS, with a main focus on the treatment of the disease flares.

## The Burden of Periodic Fever Syndromes During the Pandemic

The impact of PFS, including the Periodic Fever, Apthous Stomatitis, Pharyngitis and Cervical Adenitis (PFAPA) syndrome and the monogenic periodic fever syndromes, such as Familial Mediterranean Fever (FMF), Mevalonate Kinase Deficiency (MKD), Tumor Necrosis Factor Receptor-Associated Periodic Syndrome (TRAPS) and Cryopyrin-Associated Periodic Syndrome (CAPS) and others, can be of extreme relevance during the pandemic. In particular, the management of the disease flares in these children during this peculiar epidemiological situation is cause of major concerns. In children with PFS, the differential diagnosis between a febrile typical episode and an infection is not always immediate, as demonstrated by the rate of access to medical care (primary care physicians, emergency department, laboratory and imaging investigations) and hospitalization during the disease flares (3). Moreover, PFS carry a high cumulative number of febrile days (estimated to be 60/year in PFAPA patients), which is responsible of school absences, reduced socialization and of a significant health-related and indirect economic burden (3). Also the impact of the periodic fever syndromes on the caregivers is relevant, as emerges from a recent study on FMF, which evidences that the healthcare related burden is perceived as moderate or severe in a significant percentage of the caregivers (40%) (4). During the pandemic the effect of a febrile episode can be magnified, and children with PFS can represent a population carrying a markedly elevated risk of undergoing isolation and repetitive swabs for the detection of SARS-CoV-2 (in absence of other validated diagnostic methods), with the consequent implications, including reduced school attendance, children and parental stress and loss of days of work by the caregivers.

## The Challenges For the Clinicians

During the pandemic the clinicians, including primary care physicians and specialists, could face significant difficulties in the approach to children with PFS, in terms of diagnosis, long-term management and the treatment of the disease flares.

### Diagnosis of Periodic Fever Syndromes

Since the first stages of the pandemic it clearly emerged that the diagnosis of several chronic disorders, even in childhood, is often significantly delayed. This mostly derives from the reduced referral to primary care physicians and from the reduction of the outpatient activity in the secondary and tertiary level centers. Moreover, the concern of the potential nosocomial SARS-CoV-2 infections caused a reduced attendance to outpatient visits in many settings. The use of telemedicine services can help in the diagnostic process, since an accurate analysis of the clinical history (with a particular focus on the diary of fever and previous laboratory investigations) are the main elements for the formulation of the diagnostic suspect of PFS. However, it is important to remark that, although telemedicine can be a feasible diagnostic tool for the specialist (immunologist, rheumatologist), its application for the primary care physician is often limited in terms of both time and resources. Therefore, the difficulties in acceding to the primary care and the consequent latency in the referral to the specialists could be the leading causes of the diagnostic delay of PFS.

### Follow-Up and Specific Risk of Severe COVID-19

The follow-up of children with diagnosed PFS is less influenced by the pandemic, since telemedicine services are usually appropriate for the evaluation of the recurrence of the disease flares and their main clinical features. Furthermore, another aspect to be considered during follow-up, with significant influence on the patient's quality of life, is represented by the disease-specific measures related to COVID-19. Some concerns have been raised on the potential increased susceptibility to COVID-19 in children with rheumatic disorders, including PFS, focusing on children treated with immunosuppressive agents. However, the scientific community has not demonstrated an increased risk of SARS-CoV-2 infection or severe COVID-19 in children with PFS, including those ones treated with biologic agents, compared to general population (5). Therefore, current recommendations do not include children with PFS among the groups deserving limitation of the school attendance, with the exception of children chronically treated with high-dose corticosteroids or cyclophosphamide (6).

Interestingly, studies on FMF showed that pyrin mutations cause a resistance to Yersinia infection, suggesting that individuals with MEFV mutations could have been selected during the plague pandemics (7). Although there is currently no evidence of a protective role of mutations causing PFS against COVID-19 or other viral infections, the analysis of the complex mechanisms underlying the inflammasome activation could provide useful insights in the comprehension of the individual susceptibility to severe COVID-19.

Finally, as the measures to limit the spreading of SARS-CoV-2 cause a significant reduction in the incidence of other respiratory infections, the absence of infectious triggers could reduce the frequency of the disease flares in children with monogenic PFS, such as FMF or MKD. During the pandemic, also the changes in the pattern children's stress can have an influence on the recurrence of flares, which has to be better defined.

### Long-Term Therapeutic Management

For what concerns the therapeutic approach to children with PFS, there is no evidence that the use of immunosuppressive agents, and in particular colchicine and anti-IL-1 drugs, which represent the milestones of the chronic treatment of monogenic PFS, can increase the risk of severe COVID-19. As a consequence, it is strongly recommended to continue anti-rheumatic agents in adult and pediatric patients suffering from rheumatic diseases (8, 9). Biologic agents (anti-IL-1 and anti-IL-6 drugs) are used in the treatment of the severe manifestations of COVID-19 (10), and other anti-rheumatic drugs (including colchicine) have been proposed to prevent severe COVID-19 (11). Currently there are no data showing a potential protection against COVID-19 in children with PFS treated with anti-rheumatic agents. On the other hand, data coming from the national registries of multisystemic inflammatory syndrome in children (MIS-C) associate to COVID-19 show that the presence of an underlying rheumatic or autoinflammatory condition and/or the ongoing treatment with colchicine or anti-Il-1 drugs do not represent risk factors for the development of this condition (12).

### Management of the Disease Flare: A Practical Approach During the Pandemic

The clinical and diagnostic approach to fever in these children can represent a significant clinical challenge, as the measures to exclude SARS-CoV-2 infection and limit its spreading have to be balanced with the peculiarity of PFS, which are featured by recurrent episodes of non-infectious fever. PFAPA syndrome is the most common cause of periodic fever in pediatric age, and the features of the disease flares in the affected children could partly overlap with the typical clinical presentation of pediatric COVID-19, thus representing a difficult condition to manage in the clinical practice, especially for primary care physicians. An accurate analysis of the clinical features of the febrile episode is mandatory, with rhinorrhea, cough, unusual gastrointestinal manifestations (i.e., diarrhea), diffuse arthro-myalgia being central elements in the differential diagnosis, together with the investigation on the known exposure to SARS-CoV-2 positive patients and the prevalence rate of COVID-19 in the specific geographic area. In contrast with the monogenic recurrent fevers (FMF, MVK, TRAPS, CAPS), PFAPA patients are usually treated with non-steroidal anti-inflammatory drugs (NSAIDS) or steroids on demand and do not receive a continuous prophylactic treatment with colchicine or anti-IL-1 dugs.

Based on the evidences acquired so far, it is reasonable to suggest not performing specific investigations for the detection of SARS-CoV-2 (including the rhino-pharyngeal swab) in patients with already diagnosed PFS when the disease flare has typical clinical features without signs of respiratory or gastrointestinal infection and, in the case of PFAPA syndrome, when it is aborted or attenuated by the administration of steroid or NSAIDs on demand. This approach could significantly reduce the stress related to the single febrile episode, also limiting the negative impact of the disease flare on the school attendance. When one of these conditions is absent, the execution of the swab and the measures of isolation are necessary.

On the other hand, the management strategy can be more blurred in children with recurrent episodes of fever in which a diagnosis of periodic fever syndrome has not been formulated. In these children, performing a swab can be more frequently necessary during the episodes; nevertheless, the physician's awareness about periodic fever syndromes, the regularity of the recurrence and the clinical phenotype of the episodes and the response to a therapeutic challenge with single-dose corticosteroids (in the suspect of PFAPA syndrome) should represent important issues to be considered. At this regard, the cooperation between primary care physicians and rheumatologists is essential to accurately select patients which can be managed without undergoing into specific investigations for SARS-CoV-2 during each febrile episodes and which can benefit from the administration of corticosteroids (not recommended in the mild COVID-19) (13) during the disease flare. At this regard, it is important to underline that a single dose of corticosteroids being administered in a misdiagnosed COVID-19 patient, although not effective against the disease, is not likely to negatively influence the clinical course. Similarly, current evidences do not support the hypothesis of a potential negative effect deriving by the administration of NSAIDS in COVID-19 patients (14).

## Concluding Remarks

The diagnosis and management of children with PFS during the COVID-19 pandemic can be significantly challenging, and the approach to the disease flares is of particular relevance.

Specific indications on the matter are warranted, as during the winter season the number of children presenting with fever is expected to dramatically increase, and identifying populations deserving different clinical, diagnostic and epidemiological management can significantly contribute in the improvement of the healthcare response to the pandemic.

## Author Contributions

RC and MG conceptualized the manuscript and critically revised the initial draft. GC wrote the initial draft of the manuscript. All authors contributed to the article and approved the submitted version.

## Conflict of Interest

The authors declare that the research was conducted in the absence of any commercial or financial relationships that could be construed as a potential conflict of interest.
